# Molecular Mechanisms Involved in MAFLD in Cholecystectomized Patients: A Cohort Study

**DOI:** 10.3390/genes14101935

**Published:** 2023-10-13

**Authors:** Shreya C. Pal, Stephany M. Castillo-Castañeda, Luis E. Díaz-Orozco, Mariana M. Ramírez-Mejía, Rita Dorantes-Heredia, Rogelio Alonso-Morales, Mohammed Eslam, Frank Lammert, Nahum Méndez-Sánchez

**Affiliations:** 1 Faculty of Medicine, National Autonomous University of Mexico, Tlalpan, Mexico City 04510, Mexico; shreyacarmenpal@gmail.com (S.C.P.); mc05luisdiaz@gmail.com (L.E.D.-O.); 2 Medical, Dental and Health Sciences Master and Doctorate Program, National Autonomous University of Mexico, Tlalpan, Mexico City 04510, Mexico; smcc-fanny@hotmail.com; 3 Liver Research Unit, Medica Sur Clinic & Foundation, Tlalpan, Mexico City 14050, Mexico; mich.rm27@gmail.com (M.M.R.-M.); rdorantesh@medicasur.org.mx (R.D.-H.); 4 Plan of Combined Studies in Medicine, Faculty of Medicine, National Autonomous University of Mexico, Tlalpan, Mexico City 04510, Mexico; 5 Genetic and Molecular Biology Laboratory, Faculty of Veterinary, National Autonomous University of Mexico, Tlalpan, Mexico City 04510, Mexico; ralonsom@unam.mx; 6 Storr Liver Centre, Westmead Institute for Medical Research, Westmead Hospital, University of Sydney, Sydney, NSW 2145, Australia; mohammed.eslam@sydney.edu.au; 7 Health Sciences, Hannover Medical School (MHH), 30625 Hannover, Germany; lammert.frank@mhhannover.de

**Keywords:** cholecystectomy, fatty liver, fibrosis, FXR1, FGFR4

## Abstract

Gallstone disease and metabolic dysfunction-associated fatty liver disease (MAFLD) share numerous common risk factors and progression determinants in that they both manifest as organ-specific consequences of metabolic dysfunction. Nevertheless, the precise molecular mechanisms underlying fibrosis development in cholecystectomized MAFLD patients remain inadequately defined. This study aimed to investigate the involvement of farnesoid X receptor 1 (FXR1) and fibroblast growth factor receptor 4 (FGFR4) in the progression of fibrosis in cholecystectomized MAFLD patients. A meticulously characterized cohort of 12 patients diagnosed with MAFLD, who had undergone liver biopsies during programmed cholecystectomies, participated in this study. All enrolled patients underwent a follow-up regimen at 1, 3, and 6 months post-cholecystectomy, during which metabolic biochemical markers were assessed, along with elastography, which served as indirect indicators of fibrosis. Additionally, the hepatic expression levels of FGFR4 and FXR1 were quantified using quantitative polymerase chain reaction (qPCR). Our findings revealed a robust correlation between hepatic FGFR4 expression and various histological features, including the steatosis degree (r = 0.779, *p* = 0.023), ballooning degeneration (r = 0.764, *p* = 0.027), interphase inflammation (r = 0.756, *p* = 0.030), and steatosis activity score (SAS) (r = 0.779, *p* = 0.023). Conversely, hepatic FXR1 expression did not exhibit any significant correlations with these histological features. In conclusion, our study highlights a substantial correlation between FGFR4 expression and histological liver damage, emphasizing its potential role in lipid and glucose metabolism. These findings suggest that FGFR4 may play a crucial role in the progression of fibrosis in cholecystectomized MAFLD patients. Further research is warranted to elucidate the exact mechanisms through which FGFR4 influences metabolic dysfunction and fibrosis in this patient population.

## 1. Introduction

Gallstone disease and metabolic dysfunction-associated fatty liver disease (MAFLD) have a wide array of shared risk factors within their development [1,2,3], as well as interlinked pathologic mechanisms such as insulin resistance (IR) [4,5], an altered gut microbiome [6], oxidative stress and immune mechanisms [7,8,9,10]. The treatment of gallstone disease in many cases is the surgical removal of the gallbladder, commonly known as cholecystectomy [11]. After a cholecystectomy, a cascade of disturbances in the intricate metabolic processes of the organism is triggered [12]. One of the primary consequences of this procedure is the impairment of the enterohepatic circulation, as a result of the elimination of the rhythmic functions of the gallbladder. The absence of the regulatory functions of the gallbladder leads to an erratic metabolism and the recirculation of bile acids (BAs), affecting the key homeostatic pathways of BAs and their receptors, such as the farnesoid X receptor (FXR) [12,13]. Additionally, the fine-tuned processes regulating lipid and glucose metabolism in the liver are altered. These alterations, which occur in multiple organs such as the intestine, adipose tissue and muscle, contribute to the development of metabolic disorders, such as metabolic syndrome and MAFLD [14,15]. A recent study showed that patients with long-standing cholecystectomies (≥6 months) are at increased risk of severe liver fibrosis and cirrhosis at the time of MAFLD diagnosis, compared to patients with cholecystectomies performed recently [16]. Additionally, a meta-analysis demonstrated that cholecystectomy is associated with a 1.63 fold increase in the risk of liver disease, particularly MAFLD [12]. In non-obese Hispanic people, it has been observed that cholecystectomy is a risk factor for MAFLD and other IR-associated conditions, due to an increase in hepatic fat content, the homeostatic model assessment (HOMA) index, and apoB concentrations [17]. The regulation of triglyceride levels is determined by a varying balance between the rate of production and the clearance rate. Carbohydrate metabolism into fatty acids and triglycerides is carried out in the liver, leading to the production of very-low-density lipoproteins (VLDLs). Once in the circulation, the triglycerides within the VLDLs are broken down through the action of lipases, leading to the production of free fatty acids which are readily used as energy sources in the skeletal muscle and the heart. Whenever there is an excess of free fatty acids circulating in plasma, the liver can take up the excess and metabolize it into triglycerides, which are the intrahepatic storage form. The rate of fatty acid synthesis is modulated through the transcription of SREBP-1 and the hepatic peroxisome proliferator-activated receptor alpha (PPAR-α), among others.

Gallstones and MAFLD may develop concurrently, and their association is strongly influenced by the presence of common risk factors, such as those mentioned earlier (i.e., epigenetics, genetic variations, metabolic factors) [18]. In addition to insulin resistance (IR), other significant mechanisms contributing to metabolic dysfunction have been investigated, notably the dysfunction of FXR signaling [19]. FXR, a BA nuclear receptor found in the intestines, kidneys, and liver, plays a pivotal role as a regulator of diverse metabolic pathways involved in lipid and glucose homeostasis [20]. FXR limits fatty acid accumulation in the liver through two mechanisms: (a) promoting β-oxidation by activating the hepatic PPAR-α, and (b) through plasma very low density lipoprotein (VLDL) triglyceride clearance. Its primary function is to control BA synthesis and enterohepatic circulation, crucial processes in maintaining metabolic balance [21,22]. FXR regulates glucose balance by exerting a protective role against insulin resistance and type 2 diabetes by inhibiting gluconeogenesis and glycolysis through PEPCK phosphoenolpyruvate carboxykinase (PEPCK) and glucose-6 phosphatase inhibition. A 2006 landmark study shed light on the intricate connection between FXR and insulin signaling. Mice lacking the FXR gene exhibited impaired insulin signaling in skeletal muscle and liver, along with reduced peripheral serum glucose disposal, emphasizing the role of FXR in glucose metabolism [23]. Fibroblast growth factor receptor 4 (FGFR4) is a transmembrane receptor that plays a very important role in the regulation of hepatic bile acid and lipid metabolism [24]. Among the four FGFRs found in the liver of mature adults, FGFR4 is the only one that is exclusively expressed in mature hepatocytes [25]. In addition to its role in the aforementioned processes, a very important effect of FGFR4 can be observed in fatty liver disease development, as exemplified by the metabolic outcomes of FGFR4-deficient mice [24]. The connection between FXR and FGFR4 is intricate and finely regulated. FXR activation prompts BA synthesis and release into the ileum, leading to the increased expression of fibroblast growth factor 19 (FGF19), which in turn binds to FGFR4 [26]. The activation of FGFR-4 inhibits cholesterol 7 alpha-hydroxylase (CYP7A1), which is pivotal in the synthesis of bile acids from cholesterol, through the mitogen-activated protein kinase (MAPK) pathway, suppressing additional BA production [27,28]. What makes this regulative mechanism truly impressive is its autoregulatory nature. BAs, essential for various physiological processes such as digestion and fat absorption, exert a feedback effect on their own synthesis. Through the FXR-FGFR4 pathway, bile acids essentially regulate their own production, creating an autoregulatory loop that maintains metabolic balance [29]. The importance of these findings is amplified by the significant similarity in gene sequences between human FGFR4 and its murine equivalent, underscoring the relevance of these discoveries in translational research [30,31]. Furthermore, contributing to the vast inter-relationships within the gut-liver axis, changes in the composition of bile acids (which are metabolized by gut microbiota) consequently alter the metabolic roles and pathways regulated by BAs, including FXR expression. The use of FXR agonists has shown potential benefits in reducing liver inflammation and fibrosis in the hepatic bile acid pool. However, in order for these preliminary findings to evolve into established care for patients with steatohepatitis or any stage of MAFLD, the additional genetic pathways for each of the genes (FXR, FGFR4) must be studied and clearly elucidated [32]. The aim of this study was to explore the molecular mechanisms of MAFLD development in cholecystectomized patients, specifically the levels of FGFR4 and FXR1 expression, and whether or not there is an association with liver biopsy data, as well as with subsequent RT-qPCR analysis, immunohistochemistry and the degree of fibrosis.

## 2. Materials and Methods

We carried out the following study by analyzing 12 liver biopsies taken from Medica Sur Clinic & Foundation in Mexico City in 2021 during programmed cholecystectomies. Patients were previously diagnosed with MAFLD based on the diagnostic criteria proposed in 2020 by Eslam et al. This allowed us to evaluate the expression of the genes, FGFR4 and FXR1. A fraction of the samples also underwent immunohistochemical marking with anti-FXR1 and anti-FGFR4 antibodies for a qualitative assessment of the degree of immunostaining, for comparative purposes. The expression of these genes was determined through RT-qPCR analysis of the RNA of the biopsied liver tissues. Furthermore, all patients included within the study were seen at a 1-, 3- and 6-month follow-up appointments, where metabolic biochemical markers were measured (based on a liver function test, glucose, insulin, HbA1c, and lipid profile). We also performed an elastography for each patient as an indirect fibrosis indicator.

### 2.1. Selection of Patients

Individuals were selected according to the following inclusion criteria: patients had to have a diagnosis of MAFLD and a programmed cholecystectomy for gallbladder lithiasis and cholesterol stones. The exclusion criteria were evidence of liver cirrhosis, liver failure or hepatocellular carcinoma based on previous laboratory or imaging tests, or as determined by previous or intraoperative biopsies and cholelithiasis due to pigment stones or components other than cholesterol. The elimination criteria were loss to follow-up, the development of cirrhosis and/or cancer, severe complications of laparoscopic surgery, death of the patient and voluntary discharge.

### 2.2. RT-qPCR Method

The relative genetic expression in the liver was evaluated for the fibroblast growth factor receptor 4 (FGFR4) and the farnesoid X receptor (FXR). Two reference genes were used for the relative genetic expression evaluation and normalization: the hypoxanthine phosphoribosyltransferase 1 (HPRT1) and the hydroxymethylbilane synthase (HMBS) (see Table 1 for the gene bank accession numbers).

Total RNA was purified from the liver biopsies using the Kit Quick-RNA™ (Zymo Research, Irvine, CA, USA) following the recommended producer instructions. The RNA was quantified through spectrophotometry at 260 nm and 280 nm. The purity of the RNA was assessed using the ratio of the absorbances 260 nm/280 nm. The RNA obtained through our procedures consistently had a ratio close to 2.0. The RNA was employed in RT-qPCR assays for the assessment of the FGFR4, FXR, HPRT, and HMBS gene expression. The primers used in each of the amplification procedures are shown in Table 1. Primers were obtained from T4 Oligo (Irapuato, Mexico). The primer selection was performed using the primer 3 software (https://primer3.ut.ee/, accessed on 26 June 2023), and to maximize the specific mRNA amplification, the selected primers were placed flanking introns. The primers were validated assessing their PCR product sizes using agarose electrophoresis and Sanger nucleotide sequencing.

The RT-qPCR reactions were run in the Roto-Gene 6000 (Corbett Research–Qiagen GmbH, Mexico city, Mexico) instrument, employing a one-step RT-qPCR evagreen dye mix (Super Mix RT-qPCR 2X-EG, BioTecMol, Ciudad de México, Mexico). The amplification conditions were the following: incubation at 42 °C for 30 min, followed by 30 cycles at 94 °C, 30 s; 60 °C, 30 s; and 72 °C, 30 s, in a consecutive fashion.

The relative gene expression of FGFR4 and FXR was evaluated in reference to the HPRT and HMBS expression using the ΔΔCq method [33]. All of the experiments were duplicated in order to corroborate the obtained data. The results are presented as fold changes in the mRNA in relation to the reference genes.

### 2.3. Immunohistochemistry Methodology

The hepatic biopsies were initially processed by fixing through 10% neutral buffered formalin (NBF) for 9 h and were subsequently embedded in paraffin wax blocks. The blocks were cut using the microtome into 10 µm thick sections. For each hepatic biopsy, 3 slides were processed in order to evaluate them for differences in staining, expecting to see similar staining patterns in each of the slides for the same biopsy. They were then deparaffinized through double immersion in xylene for 10 min, with posterior progressive rehydration through ethanol immersion (5 min in 100%, 95%, 80% and 60% ethanol, respectively). The sections were rinsed with distilled water for 3 min at a time, performing the process twice; it is important to mention that the rinsing process was carried out every time a solution was added for processing; this stage of the method will not be mentioned in the rest of this section to avoid repetition. Antigen retrieval was carried out by heating the slides in a microwave for 10 min at 50% power (500 Watts) while they were submerged in citrate buffer (Citrate Buffer Solution, 0.09 M, Sigma Aldrich C2488, Naucalpan de Juarez, Mexico). After heating, they were cooled in a citrate buffer at room temperature for 30 min.

To quench endogenous peroxidase activity, the slides were incubated with 3%H_2_O_2_ solution (diluted with distilled water) for 10 min each. Protein blocking with 5% bovine serum albumin (BSA) diluted in 1× TBS for 1 h was carried out. The sections were then incubated overnight at 4 °C with the anti-FXR antibody (IgG rabbit polyclonal antibody against FXR, initial concentration of 0.35 mg/mL, GeneTex, Irvine, CA, USA. Cat No. GTX113867) as well as with FGFR4 (IgG rabbit polyclonal antibody against FGFR4, initial concentration of 0.72 mg/mL, GeneTex, Cat No. GTX134355), both diluted at 1:100 in 1× TBS, as per the antibody manufacturer’s recommended dilution.

For signal detection, the samples were incubated with a peroxidase-labeled polymer (Streptavidin—Peroxidase Polymer, Sigma Aldrich S2438, Naucalpan de Juarez, Mexico) for 30 min, with posterior application of diaminobenzidine tetrachloride as chromogen (DAB Substrate, Roche 11718096001, Naucalpan de Juarez, Mexico) for 8 min until a brown color emerged. Counterstaining was performed using hematoxylin for 30 s, and the samples were rinsed with purified drinking water. The samples were dehydrated through gradient ethanol immersion and mounted for visualization. The process was repeated with a control tissue derived from human tonsils in the hospital’s storage from previous biopsies, taken from elective tonsillectomies. The tonsil tissue was used as a staining control given that it is the control tissue recommended by the antibody manufacturers, as it presents with intense immunostaining with the studied antibodies.

### 2.4. Statistical Analysis

Statistical analyses were performed using the Mann–Whitney test for the non-normally distributed data and the Friedman test for the comparison between the analytes from the basal level to the 1-, 3- and 6-month follow-ups, with *p* values <0.05 considered significant. All data are presented as the median and interquartile range. Spearman’s test was performed to calculate correlations, and the Benjamini-Hochberg method was used to correct the *p* value. We calculated the Ct comparative real-time PCR with efficiency correction using the Pfaffl equation. All analyses and graphs were created using SPSS 26.0 (SPSS Inc., Chicago, IL, USA) for Windows.

## 3. Results

### 3.1. Baseline Characteristics

This study included a total of 12 participants, with 16.7% (n = 2) males and 83.3% (n = 10) females, with a mean age of 48 ± 13 years. In total, 58.3% (n = 7) of these individuals had a MAFLD diagnosis at the time of the study. The baseline characteristics of the subjects are given in Table 2.

### 3.2. Immunologic Analysis of FXR1 and FGFR4 in Liver Biopsies

**Figure 1:**
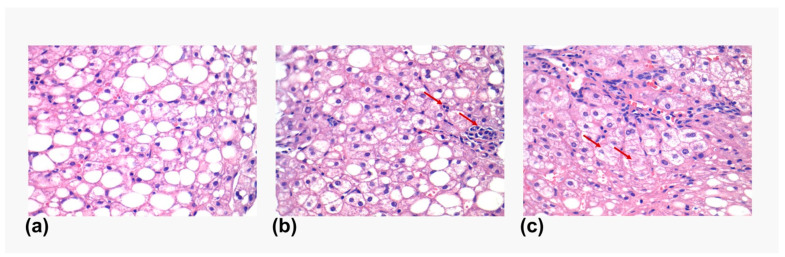
Histology features in liver biopsies. Representative images showing the different histological features observed in the liver biopsies. (**a**) Steatosis. (**b**) Lobular inflammation (marked with a red arrow). (**c**) Ballooning degeneration (marked with a red arrow).

**Figure 2:**
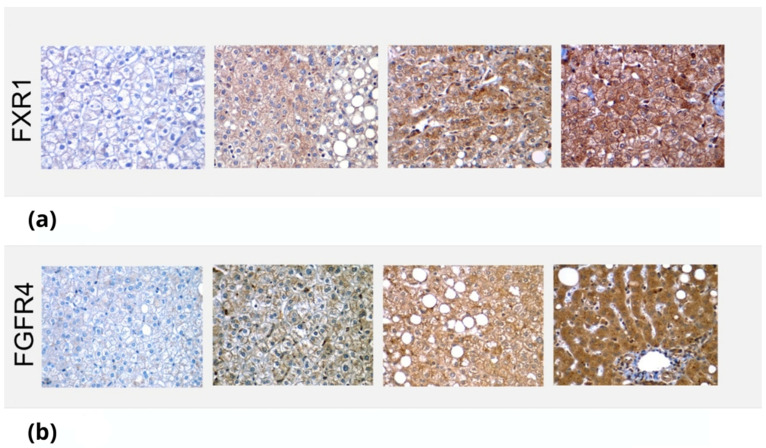
Immunostaining of FXR1 and FGFR4 in liver biopsies. The images depict the differential staining in liver biopsies, which were then qualitatively marked, depending on intensity, as +, ++ or +++. (**a**) Immunostaining of FXR1 on liver biopsies of different patients. The images show no staining (0), mild (+), moderate (++) and intense (+++) staining, respectively. (**b**) Immunostaining of FGFRA on liver biopsies of different patients. The images show no staining (0) and mild (+), moderate (++) and intense (+++) staining, respectively.

The slides were visualized and initially assessed for the steatosis degree, ballooning degeneration, and interphase inflammation and were given a steatosis activity score (SAS) (Figure 1). Afterwards, they were visualized to assess the degree of immunostaining, which was performed qualitatively, marking them with +, ++ or +++ according to the staining intensity and area evaluated by the expert pathologist (Figure 2).

### 3.3. RT-qPCR Results for Liver Biopsies

The RT-qPCR results for each of the 12 liver biopsies are shown in Table 3 for FXR1 and Table 4 for FGFR4. There was a strong correlation between the expression of FGFR4 and steatosis degree (r = −0.642, *p* = 0.033), as well as a correlation with interphase inflammation (r = −0.671, *p* = 0.024) (Supplementary Table S1). Furthermore, FXR1 expression through RT-qPCR did not have any significant association with any type of histological hepatocyte injury. The immunological marking did not show any regional differences from one biopsy to another.

### 3.4. 6-Month Follow-Up

Serum triglycerides were significantly different between patients with steatosis and those with steatohepatitis (*p* = 0.048), as well as serum insulin levels; however, other metabolic biochemical markers remained without significant change between patients before cholecystectomy and in the 6-month follow-up period (Supplementary Table S2). Among the participants, one of the patients progressed in the degree of fibrosis after the cholecystectomy (from F1 to F2) according to the transient elastography.

## 4. Discussion

**Figure 3:**
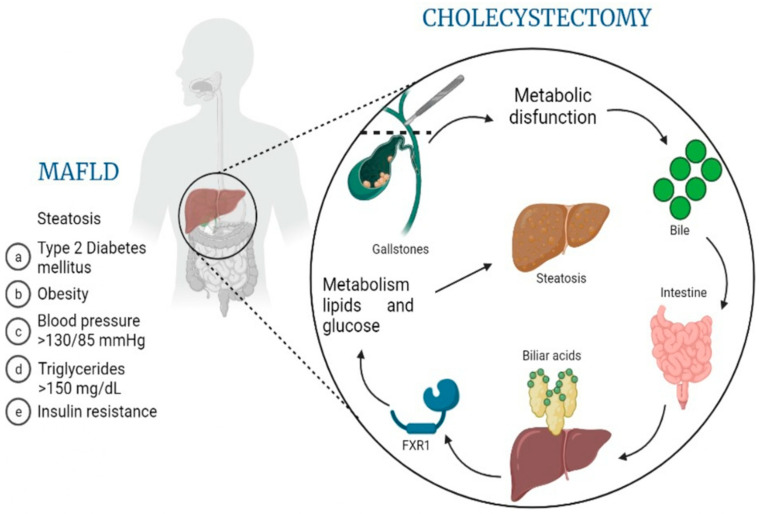
MAFLD pathogenesis related to molecular changes after cholecystectomy. This overview image hypothesizes the role that cholecystectomy might have on the development or progression of MAFLD (metabolic dysfunction-associated fatty liver disease), based on the release of biliary acids, their processing by the intestine, the consequent autoregulation of bile acid synthesis through the downregulation of FXR1 and, finally, altered lipid and glucose metabolism leading to worsening steatosis and disease progression.

Through the analysis of previous studies and reviews, we have discussed the association between cholelithiasis and MAFLD, specifically what happens in patients who have undergone a cholecystectomy [2,7,8,9,16,34]. Figure 3 illustrates our hypothesis about the possible roles of FXR1 and FGFR4 in MAFLD pathogenesis and progression. The present study allowed us to explore the mechanisms involved in this association in greater depth. Before we start with the discussion of our results, we should establish some of the baseline expectations we had before carrying out this study. All patients presented with MAFLD at the moment of cholecystectomy (diagnosed with the 2020 MAFLD criteria [35]); therefore, elevated FGFR4 levels at the time of biopsy obtainment would be expected, according to the existing literature [36,37]. FXR levels were anticipated to be low compared to healthy patients for two reasons: (a) the patients underwent cholecystectomy due to cholestasis or, in some cases, cholecystitis, which results in a very high chance of FXR being less expressed, as shown in various studies featuring FXR in cholestatic disease [38]; (b) low FXR expression is associated with metabolic dysregulation regarding lipid and glucose metabolism, both processes being altered in MAFLD [39]. When analyzing the data, we observe two findings which coincide with our hypotheses. Firstly, there was a statistically significant association between the expression of FGFR4 in the hepatocytes and the histological measurements for fatty liver (i.e., the steatosis degree, ballooning degeneration, interphase inflammation, etc.). This was expected and further strengthens our pre-existing beliefs about the role of FGFR4 in fatty liver development. In this case, the anticipated elevated levels of FGFR4 would occur independent of the temporal association with the cholecystectomy in that the direct relationship associated with increased gene expression is the relationship with fatty liver, not necessarily that with changes in the liver associated with cholecystectomy. As mentioned in the Introduction, increased FGFR4 is associated with a higher degree of hepatic steatosis [37,40]. There was no association between FGFR4 expression and the progression to fibrosis in the patients, which, as we already mentioned, only occurred in 1 out of the 12 patients. However, the gene expression was measured using the liver biopsies at the time of cholecystectomy, making it impossible to determine whether or not the expression of either gene changed after the removal of the gallbladder and whether this contributed to the progression of fibrosis.

It should also be noted that the only patient who progressed in their liver fibrosis stage during the follow-up measurements was the person who had the worst metabolic profile (i.e., weight, BMI, biochemical parameters, etc.) before the cholecystectomy took place, which might indicate a greater importance of pre-cholecystectomy somatometric and metabolic parameters when considering the factors involved in MAFLD progression and fibrosis development.

The absence of an association between FXR expression and biopsy findings of liver damage does not confirm nor reject the possible role of this gene in MAFLD genesis or progression, given the lack of a posterior liver biopsy analysis, which would be necessary to evaluate changes in FXR expression.

The same rationale can be highlighted regarding FGFR4 and its association with MAFLD progression to fibrosis or changes in the metabolic profiles of the patients. The immunohistochemical marking had the purpose of qualitatively assessing the expression of FGFR4 and FXR1 in the liver biopsies to understand how the stain intensity could be associated with differential results regarding the RT-qPCR expression levels. There was a positive association between the intensity of the staining and the levels of the genes expressed and measured using RT-qPCR. We expected to see a difference in the areas which were marked (i.e., periportal, pericentral, etc.). However, the histological data did not show any regional differences. Given that fibrosis is initially triggered by Ito cells, which are located within the perisinusoidal space, we would expect increased staining within the periportal region.

The main limitation of the study was that we were not able to take liver biopsies from the same patients after the 6-month period in order to observe the changes in gene expression, which would have better guided us toward an assessment of their role in liver structural changes after cholecystectomy. Secondly, given the observational nature of this study, the sample size was limited. Increasing the number of participants would undoubtedly provide us with data with more statistical significance and clearer associations among the measured parameters (e.g., lipid profile, degree of fibrosis as per hepatic elastography measurement, etc.) [41]. Finally, extending the follow-up period to at least 1 year could yield more significant data, given that fibrosis does not develop at the same rate in all patients and is a chronic and progressive issue.

## 5. Conclusions

The progression of fibrosis after cholecystectomy was not associated with increased FGFR4 expression, but rather with a significantly worse metabolic profile at the moment of cholecystectomy. We found a strong correlation linking FGFR4 expression with ballooning degeneration, interphase inflammation, and SAS, pointing toward its role in lipid and glucose metabolism. After cholecystectomy, there was no progression in fibrosis within 6 months, although there was an increase in the degree of steatosis. These preliminary results could guide future research by highlighting the role that these genes might have in the metabolic profiles of patients with MAFLD.

## Figures and Tables

**Table 1:** Primers used in the RT-qPCR gene expression evaluation.

Gene—Genbank Entry Primers	Exon Location Primer Sequences	Amplicon Size bp
FGFR4 NM_002011	13–14	180
FGFR4-F	tgctggtgactgaggacaat	
FGFR4-R	ggatcccaaaagaccacacg	
FXR NM_001206979	8–9	250
FXR-F	cgacaagtgacctcgacaac	
FXR-R	ggtccaaagtctgaaatcctgg	
HPRT NM_000194	1–3	176
HPRT-F	cctggcgtcgtgattagtga	
HPRT-R	gctacaatgtgatggcctcc	
HMBS NM_001024382	1–2	233
HMBS-F	gatgagagtgattcgcgtgg	
HMBS-R	gaacaaccaggtccacttca	

This table presents the data of gene-specific amplifications based on RT-qPCR that were used for the evaluation of gene expression.

**Table 2:** Clinical, biochemical and histological characteristics at the baseline.

Characteristic	Total (N = 12)
Age (years)	48 (33–56)
BMI (kg/m^2^)	29.47 (21.22–40.62)
Patients with liver steatosis	
Grade 0	6 (50%)
Grade 1	1 (8.33%)
Grade 2	3 (25%)
Grade 3	2 (16.66%)
Patients with liver fibrosis	
Grade 1	9 (75%)
Grade 2	3 (3%)
Bilirubin (mg/dL)	0.81 (0.58–1.52)
ALT (U/L)	36.00 (18.50–51.75)
AST (U/L)	29 (16.25–34.25)
GGT (U/L)	28.50 (14.00–131.00)
ALP (U/dL)	70.00 (50.00–101.75)
Albumin (g/L)	3.92 (3.72–4.21)
Cholesterol (mg/dL)	174.10 (146.25–191.00)
Cholesterol HDL (mg/dL)	39.10 (36.25–48.65)
Cholesterol LDL (mg/dL)	111.50 (87.25–128.00)
Triglycerides (mg/dL)	94.71 (76.88–127.04)
Glucose (mg/dL)	100.80 (85.82–120.15)
HbA1c (%)	5.50 (5.25–5.75)
Insulin (μIU/mL)	7.60 (4.90–11.60)

This table summarizes the baseline characteristics of the individuals included in the study. ALP: alkaline phosphatase, ALT: alanine transaminase, AST: aspartate aminotransferase, GGT: γ-glutamyl transferase, BMI: body mass index, HbA1c: glycated hemoglobin.

**Table 3:** Relative expression of the FXR-1 gene through RT-qPCR.

Efficiency	1.54	1.67	1.605	1.68					
		Control	28.55	33.06	dCt (Target)	E (dCt Target)	dCt (hkg)	E (dCt hkg)	Ratio
Sample	HPRT	HMBS	MG HK	FXR					
1	29.85	27.24	28.55	33.06	0.00	1.00	0.00	1.00	1.00
2	26.84	24.75	25.80	32.54	0.52	1.31	2.76	3.68	0.36
3	16.67	15.90	16.29	18.18	14.88	2252.17	12.27	331.25	6.80
4	15.52	14.74	15.13	16.68	16.38	4904.18	13.42	572.10	8.57
5	15.70	14.78	15.24	16.51	16.55	5356.35	13.31	543.09	9.86
6	15.41	14.65	15.03	16.14	16.92	6489.83	13.52	599.82	10.82
7	15.38	14.45	14.92	16.26	16.80	6098.12	13.64	633.36	9.63
8	18.20	17.93	18.07	20.25	12.81	769.51	10.49	142.69	5.39
9	19.19	18.19	18.69	19.17	13.89	1347.55	9.86	106.17	12.69
10	22.52	20.69	21.61	23.36	9.80	161.45	6.95	26.73	6.04
11	22.11	20.76	21.44	23.04	10.02	180.97	7.12	28.97	6.25
12	21.86	20.56	21.21	23.17	9.89	169.16	7.34	32.22	5.25

This table presents detailed data related to gene expression analysis using RT-qPCR, including measurements of expression levels, amplification efficiency, and comparisons between HPRT, HMBS, MG HK and FXR.

**Table 4:** Relative expression of the FGFR4 gene through RT-qPCR.

Efficiency	1.54	1.67	1.605	1.68					
		Control	28.55	27.66	dCt (Target)	E (dCt Target)	dCt (hkg)	E (dCt hkg)	Ratio
Sample	HPRT	HMBS	MG HK	FGFR					
1	29.85	27.24	28.55	27.66	0.00	1.00	0.00	1.00	1.00
2	26.84	24.75	25.80	27.64	0.02	1.01	2.76	3.68	0.27
3	16.67	15.90	16.29	20.44	7.22	38.83	12.27	331.25	0.12
4	15.52	14.74	15.13	18.68	8.98	94.75	13.42	572.10	0.17
5	15.70	14.78	15.24	17.81	9.85	147.25	13.31	543.09	0.27
6	15.41	14.65	15.03	19.32	8.34	68.50	13.52	599.82	0.11
7	15.38	14.45	14.92	18.57	9.09	100.18	13.64	633.36	0.16
8	18.20	17.93	18.07	21.79	5.87	19.59	10.49	142.69	0.14
9	19.19	18.19	18.69	22.21	5.45	15.83	9.86	106.17	0.15
10	22.52	20.69	21.61	22.51	5.15	13.60	6.95	26.73	0.51
11	22.11	20.76	21.44	22.90	4.76	11.16	7.12	28.97	0.39
12	21.86	20.56	21.21	23.88	3.78	6.79	7.34	32.22	0.21

This table provides detailed data related to gene expression analysis using RT-qPCR, including measurements of expression levels, amplification efficiency, and comparisons between HPRT, HMBS, MG HK and FGFR.

## Data Availability

The data presented in this study are available in the article and in supplementary material.

## Supplementary Materials

The following supporting information can be downloaded at: https://www.mdpi.com/article/10.3390/genes14101935/s1, Table S1. Correlation of RT-qPCR results and biopsy variables. Table S2. Baseline and month follow-up results of laboratory tests.
