# Supplementary material for: Molecular Mechanisms Involved in MAFLD in Cholecystectomized Patients: A Cohort Study
Source: Genes (Basel). 2023 Oct 13;14(10):1935. doi: 10.3390/genes14101935 (PMC10606482; doi:10.3390/genes14101935)
Supplement: Supplementary file 1 [file genes-14-01935-s001.zip › genes-2568503-supplementary.pdf]

Table S1. Correlation of RT-qPCR results and biopsy variables.

	RT-qPCR FGFR4		RT-qPCR FXR1	
	**r**	***p***	**r**	***p***
Degree of steatosis	−0.642 *	**0.033**	0.241	0.475
Percentage of steatosis	−0.542	0.132	0.376	0.319
Balonoid degeneration	−0.228	0.501	−0.355	0.284
Degree of lobular inflammation	0.072	0.832	−0.550	0.079
Portal inflammation	−0.231	0.495	−0.115	0.736
Interphase inflammation	−0.671 *	**0.024**	0.149	0.662
Activity score	−0.445	0.170	−0.308	0.357
Degree of fibrosis	−0.323	0.333	−0.323	0.333

Table S1 provides the corrrelation values between RT-qPCR FGFR4 and RT-qPCR FXR with the different histological parameters evaluated. Correlation coefficients: Spearman's rho. * Correlation is significant at the 0.05 level (bilateral).

Table S2. Baseline and month follow-up results of laboratory tests.

	Baseline	1 Month	3 Months	6 Months	p
Total bilirubin (mg/dL)	0.81 (0.58–1.52)	0.76 (0.50–1.3275)	0.71 (0.54–0.91)	0.77 (0.52–0.99)	0.915
Direct bilirubin (mg/dL)	0.12 (0.10–0.84)	0.17 (0.10–0.31)	0.10 (0.10–0.14)	0.10 (0.10–0.15)	0.292
Indirect bilirubin (mg/dL)	0.68 (0.47–0.97)	0.66 (0.38–1.00)	0.61 (0.40–0.77)	0.60 (0.40–0.86)	0.706
ALT (U/L)	36.00 (18.50–51.75)	27.00 (18.50–48.00)	22.00 (18.50–29.00)	19.00 (16.00–39.00)	0.843
AST (U/L)	29 (16.25–34.25)	28.00 (19.00–39.00)	23.00 (18.50–27.50)	22.00 (20.00–28.00)	0.763
GGT (U/L)	28.50 (14.00–131.00)	35.00 (28.50–57.45)	17.00 (12.00–27.50)	14.00 (13.00–39.00)	**0.009**
FA (U/L)	70.00 (50.00–101.75)	73.50 (57.00–87.00)	62.00 (57.50–83.50)	68.00 (62.00–70.00)	0.753
Albumin (g/dL)	3.92 (3.72–4.21)	4.14 (3.82–4.40)	3.97 (3.80–4.15)	4.01 (3.86–4.20)	0.457
Cholesterol (mg/dL)	174.10 (146.25–191.00)	186.70 (156.70–221.80)	177.35 (161.85–210.42)	173.00 (145.00–214.00)	0.172
LDL cholesterol (mg/dL)	111.50 (87.25–128.00)	116.00 (95.00–147.90)	115.00 (92.50–138.50)	109.00 (79.00–136.00)	0.067
Triglycerides (mg/dL)	94.71 (76.88–127.04)	129.00 (75.36–232.62)	106.67 (90.06–115.83)	101.94 (57.43–132.00)	0.346
HDL cholesterol (mg/dL)	39.10 (36.25–48.65)	42.10 (35.50–49.35)	45.15 (38.90–56.52)	53.70 (38.00–61.70)	0.594
Glucose (mg/dL)	100.80 (85.82–120.15)	95.35 (91.75–97.85)	95.20 (90.22–105.05)	89.50 (85.90–101.00)	0.619
HbA1c (%)	5.50 (5.25–5.75)	5.40 (5.15–5.60)	5.30 (5.10–5.70)	5.30 (5.00–5.60)	0.340
Insulin (mU/L)	7.60 (4.90–11.60)	7.60 (6.10–18.95)	9.20 (5.65–12.35)	8.20 (3.20–12.00)	0.910
HOMA-IR	2.10 (0.70–2.60)	2.10 (1.39–3.74)	1.41 (0.56–2.44)	0.37 (0.38–2.32)	0.071

Table S2 provides a summary of the variation in biochemical variables during the follow-up periods. Medians, interquartile range. Friedman test, significance level is 0.050.
